# Chicago Medical Society

**Published:** 1867-09

**Authors:** 


### Proceedings of Societies,

### CHICAGO MEDICAL SOCIETY.

### SHELL WOUND OF THE HEAD.

Dr. Dunn reported an exceedingly interesting case of injury to the skull, produced by a fragment of shell.

The patient, a surgeon, while on duty during battle received the injury over the inner circle of the left eye. It was quite extensive, implicating not only the soft tissues, but also the bone beneath. There was complete insensibility for several hours. In due time the wound healed, with the exception of a small fistulous opening, leading to carious bone.

The patient was subject for two years to frequent paroxysms of terrible neuralgic pain in the head, numbness of the integument, with a sense of stricture aronnd the forehead. There was at times a slight discharge of purulent matter from the nostrils.

Although the patient has suffered much, and has at times been incapacitated for mental or physical labor, he has yet devoted himself to his professional duties.

Some weeks ago a few small fragments of bone escaped through the nostril, since which time the painful symptoms have entirely subsided.

